# Making Full Use of Qualitative Data to Generate New Fish Product Ideas through Co-Creation with Consumers: A Methodological Approach

**DOI:** 10.3390/foods11152287

**Published:** 2022-07-31

**Authors:** Laura López-Mas, Anna Claret, Violeta Stancu, Karen Brunsø, Irene Peral, Elena Santa Cruz, Athanasios Krystallis, Luis Guerrero

**Affiliations:** 1 Food Quality and Technology, Institute of Agrifood Research and Technology (IRTA), Finca Camps i Armet, s/n, 17121 Monells, Spain; laura.lopezm@irta.cat (L.L.-M.); anna.claret@irta.cat (A.C.); 2 Department of Agri-Food Engineering and Biotechnology (DEAB), Baix Llobregat Campus, Universitat Politècnica de Catalunya (UPC), Building D4, st/Esteve Terradas, 8, 08860 Castelldefels, Spain; 3 MAPP Centre, Department of Management, Aarhus BSS, Aarhus University (AU), Fuglesangs Allé 4, 8210 Aarhus, Denmark; viost@mgmt.au.dk (V.S.); kab@mgmt.au.dk (K.B.); 4 AZTI, Food Research, Basque Research and Technology Alliance (BRTA), Parque Tecnológico de Bizkaia, Astondo Bidea, Edificio 609, 48160 Derio, Spain; iperal@azti.es (I.P.); esantacruz@azti.es (E.S.C.); 5 Centre of Excellence in Food, Tourism and Leisure, American College of Greece (ACG), Gravias 6, 15342 Athens, Greece; akrystallis@acg.edu

**Keywords:** focus group, new product development, aquaculture, seafood, content analysis, pseudo-triangulation, context, co-occurrence, word frequency

## Abstract

Co-creation is a process that directly involves different stakeholders in the idea generation phase of a new product development process. A pool of 112 new aquaculture fish product ideas was obtained by applying a combination of creative and projective techniques to the co-creation process with consumers in six focus groups conducted in three European countries (Germany, France, and Spain). The subjectivity of qualitative data analysis (e.g., focus groups) is one of its recognised disadvantages. To overcome this drawback, a combination of specialised software (i.e., Alceste), along with word frequency, co-occurrence, and context checking, was applied to provide a different approach to data analyses in qualitative studies. The method identified the most salient dimensions behind the participants’ discourse (naturalness, quality, ethics, price, and health) and inferred the importance those dimensions had for them, thus proving the existence of a correlation of 0.7 between what the participants said (frequency of mention) and what they liked the most (importance). Overall, the exploratory approach proposed is deemed useful for drawing key conclusions from qualitative research, thus offering an alternative to traditional content analysis. In future, the results obtained may be useful for selecting the co-created ideas with the greatest potential to be well received in the market.

## 1. Introduction

Approximately 80% of new food products launched in the market fail within the first year [1]. One of the most effective ways to enhance new product success in the market is to actively involve end-users during the new product development (NPD) process [2,3]. The early stages of NPD are crucial because failure at these stages is inexpensive compared with the cost of launching an unsuccessful product on the market [2,3]. Idea generation, screening, and the selection of the most promising ones are usually at the beginning of any NPD procedure [4]. Co-creation is a process that directly involves different stakeholders, such as consumers, in idea generation. According to Prahalad and Ramaswamy [5], co-creation can be defined as the “joint creation of value by the company and the customer, allowing the customer to co-construct the service experience to suit her context”. The collaboration between companies and consumers through co-creation not only allows the creation of customer value [6,7], but also examines consumers’ wants and needs [8], enhances NPD performance and reduces the risk of failing to meet consumer demands [9], reduces product development costs [10], and elicits more original and valuable ideas than those created by professional developers [11]. Co-creation can be applied in traditional creative techniques (e.g., brainstorming), in which the main goal is to generate a pool of ideas in a process that is primarily cognitive [12]. In contrast to creative techniques, projective techniques are based on the principle that people’s unconscious desires can be inferred by using ambiguous and unstructured stimuli, in which the subjects project their beliefs, attitudes, feelings, and motivations [13]. The more ambiguous and unstructured the stimuli, the more consumers may reveal their underlying personalities [3,13]. Banović et al. [14] demonstrated that a combination of creative and projective techniques is a useful approach for generating new ideas in which consumers’ voices are widely incorporated. This combination of techniques can be applied to traditional qualitative methods, such as focus groups.

Focus groups are often used to capture consumers’ spoken needs [8], gain insights into their behaviour, attitudes, needs, and wants, understand the underlying motives of their choices, generate new products, and accelerate NPD processes [15,16]. The traditional semantic analysis of focus group results involves the transcription of the discussion to draw main conclusions [17], a process that can be improved through the so-called “pseudo-triangulation” [16]. Pseudo-triangulation is based on the independent analysis of three researchers or experts, reaching a consensus on a specific interpretation of the findings, which allows for the extraction of key conclusions [18]. Although the independent analysis of three researchers aims to balance the subjective influences of individuals [19], when interpreting qualitative data, it is common for a confirmatory bias to occur [20]; that is, researchers more strongly support the hypotheses that confirm what they believe [21]. Therefore, the subjectivity of the interpretation of results is one of the recognised disadvantages of qualitative techniques [22,23].

To overcome some of the limitations of traditional content analysis and manual coding, other alternatives can be used to increase the objectivity of the interpretation of results, such as the use of specialised software [24] (e.g., Alceste, ATLAS.ti, MAXQDA, NVivo). Software assistance is extremely useful during content analysis, as it allows for a reduction in content to take place in an objective way, saving time, cost, and effort [17,22]. In dealing with qualitative data, the less redundant the information there is, the easier the subsequent analysis is. Word frequency count and co-occurrent words are common outputs of qualitative data analysis software. Co-occurrence analysis reveals the relationship between words based on the number of times they are mentioned together [25], giving structure to the data.

In contrast, word frequency counting is probably the most widespread software-assisted method, as it is a simple and rapid way to summarise the results in terms of the most frequently mentioned words [26]. Hence, word frequency counting is commonly used in qualitative research as, for example, an indicator of the importance of certain words for participants [27,28,29,30]. Nevertheless, one of the main drawbacks associated with frequency counting is the loss of the context in which words are mentioned, which may lead to erroneous conclusions [17,31,32]. Therefore, because the meanings of words are often context-dependent, carrying out an in-depth qualitative analysis requires the interpretation of each word in its respective context [17].

In general, there is a lack of consensus on how to analyse and interpret qualitative data [17]. Most studies found in the literature seldom provide details on how they conducted the qualitative analysis or the complete set of codes applied [20]; some only mention “content analysis”. In addition, despite its advantages, the use of software for qualitative data analysis is still relatively limited in terms of studying the latent meaning of discourse [24]. Therefore, it could be hypothesised that the combination of software, along with the use of word frequency, co-occurrence, and context checking, could provide a different approach for data analyses in qualitative studies. The establishment of solid methodological guidelines can be extremely useful when analysing qualitative data, as it has been criticised for its rather subjective nature for a long time.

To prove the effectiveness of the methodology proposed in this study, the aquaculture fish sector was targeted. Food lifestyles are changing in Europe, and consumers usually have less time to spend on food preparation. New lifestyles, along with higher consumer awareness, have caused an increasing demand for a year-round supply of innovative and disruptive food products [33]. However, although the current food market seems to be saturated for most food categories [23], there are fewer new processed fish products in the market compared with other industries, such as meat [34], which leaves room for NPD, co-creation, and an exploration of the usefulness of qualitative data and its full potential.

Accordingly, the aim of this exploratory study is threefold: (1) to propose an alternative approach to traditional qualitative content analysis by combining word frequency, co-occurrence and contextual analysis; (2) to explore the usefulness of the proposed approach in generating aquaculture fish product ideas and identify the most relevant product dimensions affecting potential acceptance by consumers; and (3) to explore whether word frequencies can be related to the subjacent relevant concepts or dimensions for the participants involved.

## 2. Materials and Methods

### 2.1. Participants’ Recruitment

Purposive sampling with a predetermined quota for gender (evenly split) was used to select 36 participants in three countries (France, Germany, and Spain). A market research agency based in the three countries under study was subcontracted in June 2019 to recruit the participants and lead the moderation of the focus groups. Country selection was based on various aspects that may influence the generation of distinct ideas of fish products, including: differences in fish consumption per capita (Spain > France > Germany) [35]; differences in the main place of fish purchase—grocery store (Germany) or fishmonger/market (Spain) [36]; and the number of new fish products available in the national markets (France > Germany > Spain) [34].

In addition, participants also met the criteria of being older than 18 years, responsible for food purchase and preparation within their household, and fish consumers. In each country, fish consumption was used to divide participants into two groups: regular (at least once a week) and occasional fish consumers (three times a month or less), as it can be assumed that fish consumption frequency may be related to the demand for different fish products.

### 2.2. Focus Group Sessions

**Figure 1:**
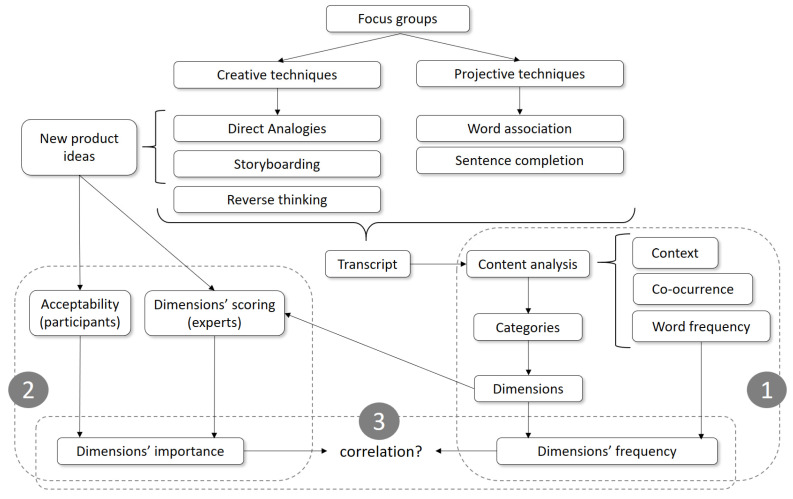
Outline of the main steps taken during data analysis of the focus groups: (**1**) dimensions’ identification and their frequencies; (**2**) dimensions’ importance; and (**3**) dimensions’ importance versus dimensions’ frequency.

Two face-to-face focus groups were conducted in each of the three countries targeted. Each focus group session with six participants lasted for two hours. The relatively low number of participants per focus group was selected based on the criteria that qualitative research does not intend to make inferences to a larger population, but to gain a deeper understanding of consumers’ perceptions and opinions about fish products [16]. Additionally, the qualitative approach used allowed participants to generate ideas about new aquaculture fish products. The moderators from the market research agency were previously briefed and followed a detailed discussion guide that included the following sections (Figure 1):

1. A warm-up debate about new foods and fish products.
2. A creative approach by applying direct analogies (also known as analogical thinking) [37] and storyboarding [14] to engage participants in generating new product ideas. Additionally, a reverse thinking task (also known as reverse brainstorming, tear-down, or purge) [38] was included to determine which characteristics of the ideas previously elicited would be rejected by the rest of the consumers.
3. A section where participants scored their acceptance of each new product idea elicited in the creative phase on a scale from 1 (“I very much dislike this fish product idea”) to 10 (“I like this fish product idea”).
4. A projective approach that included word association [13,14,18,39] and sentence completion tasks [13,39]. Four new fish product concepts identified by Gartzia et al. [40] were used as stimuli in both projective tasks to gain useful insights into consumers’ latent desires and feelings.
5. Finally, a general discussion section about fish and fish products to gain a deeper understanding of people’s practices regarding their purchase and consumption, opinions, interests, motivations, barriers, and behaviour with regard to new products.

In addition, a brief discussion of salient ideas was carried out after each task to further understand the participants’ perceptions. The above-described focus group sessions were conducted in the native language of each country, audio-recorded and videotaped, simultaneously translated into English, and transcribed verbatim for further analysis from the English translation.

### 2.3. Transcript Preparation: Data Preparation

Prior to data analysis, the transcripts were reviewed to detect and correct possible mistakes. Three researchers who were native French, German, or Spanish speakers listened to the recordings in their national languages and checked the English transcripts. Afterwards, all English transcripts were reviewed by the same researcher, following the guidelines proposed by Dalud-Vincent [41]. The spelling was standardised into British English (e.g., flavour instead of flavor) and the same spelling was used to designate the same concept (e.g., fillet instead of filet, the counterpart French word). The moderators’ speeches were removed from the transcripts, and only the participants’ individual speeches were retained. Finally, the content that was not relevant to the study was also removed (e.g., personal conversations between participants).

### 2.4. Data Analysis

Data analysis was divided into three main parts: (1) dimensions’ identification and their frequencies; (2) dimensions’ importance; and (3) dimensions’ importance versus dimensions’ frequency. All steps taken are illustrated in Figure 1 and are described in this section.

#### 2.4.1. Part 1: Dimensions’ Identification and Their Frequencies

**Figure 2:**
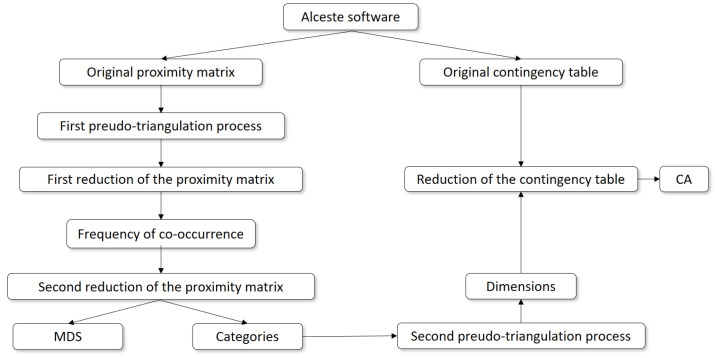
Outline of the steps taken for the identification of the categories and dimensions and their frequencies. CA: correspondence analysis; MDS: multidimensional scaling.

The final corpus included the transcripts from all countries, arranged according to IMAGE [42], allowing the Alceste software, version 2018 (2018) (IMAGE, Toulouse, France) to perform content analysis in the following stages [24,41]: (1) text segmentation: into elementary context units (ECUs), i.e., basic analysable statistical units; (2) lemmatisation: simplifying words to their lemmas, i.e., to root forms that can be found in a dictionary (e.g., plurals into singulars); (3) reduction: discarding certain words (i.e., conjunctions, prepositions, pronouns); and (4) classification: the software’s internal dictionaries serve to sort the “content words”, i.e., words that were retained, by grammatical category (nouns, verbs, adjectives, and adverbs). An example of a quotation from one of the focus groups, “I buy new products to try out new flavours”, may be used to illustrate how Alceste reduces the content into two verbs (buy, try), one adverb twice (new), and two nouns (product, flavour), while all other components are discarded (I, to, out). Alceste tabulated some of the content analyses conducted; for example, a proximity matrix (co-occurrence within an ECU) and a contingency table (word frequency). Both outputs were modified according to the steps described in the next paragraphs, as illustrated in Figure 2.

The original proximity matrix provided by Alceste had the same codes (words) in the rows and columns, while the cells were filled in with the frequency of co-occurrence (a measure of similarity), similar to a correlation matrix, but for categorical data [43]. Nevertheless, as Alceste does not consider the context in which words are said, a manual merging was conducted through the first pseudo-triangulation process with three independent researchers [18] to reduce the content. As a rule, throughout context checking in ECUs, when a word was used more than 75% of the time with a specific meaning, that meaning was attributed to that word. The reduction process through the first pseudo-triangulation consisted of the following stages:

1. Words with a common root (e.g., try, tried, and trying) were grouped under the same label (e.g., “try”).
2. Filler words, meaningless words, or sounds that consumers use while talking to fill in the pauses (e.g., basically, just, well) were removed after context checking.
3. Some words were grouped after checking their context in semantic (i.e., a set of words with related meanings) and associative semantic fields (i.e., similar to semantic fields but more subjective associations) to retain the information contained in the less frequently mentioned words.
4. Antonym words were grouped because they were considered to be extremes of the same scale (e.g., the “easy” label grouped the words easy, ease, difficult, and complicated).
5. Finally, homograph words, those with the same spelling but different meanings, were merged into their corresponding groups according to their meanings after context checking.

Once the context was checked, the original proximity matrix provided by the Alceste software was reduced accordingly, thus grouping some words and removing others.

Subsequently, a second reduction based on the frequency of mention was conducted, retaining only those words whose diagonal values in the proximity matrix were greater than 50. This cut-off was set by averaging all the values on the diagonal of the proximity matrix, rounding upward; therefore, only retaining words that co-occurred more frequently than the average. The words retained were labelled “categories”. The reduced proximity matrix was submitted to multidimensional scaling (MDS) analysis to plot the strength of the connections between co-occurring categories.

Finally, some of the results from the MDS were used to group the categories into dimensions through a second pseudo-triangulation process. The selection of the final dimensions was agreed upon by the three researches, who identified them based on the categories obtained, using input from the single-item food choice questionnaire (FCQ) [44]. The FCQ, originally developed by Steptoe et al. [45], is regarded as one of the most widespread methods used in consumer research to assess the motivations underlying food selection by measuring nine different factors.

The original contingency table provided by Alceste with the word frequency count was reduced according to the process described in Figure 2. The words elicited (rows) were grouped according to the identified dimensions by simply adding the corresponding frequencies for each participant (columns). In addition, supplementary variables (columns) were added (i.e., country, focus group session, age category, gender, and fish consumption). A simple correspondence analysis (CA) was run to graphically display the reduced contingency table.

#### 2.4.2. Part 2: Dimensions’ Importance

The importance of dimensions was calculated by multiplying the acceptability given by participants with the scores given by a group of experts (see Table 1 for an example). In detail, on one hand, the individual acceptability of the participants given to each idea generated using creative techniques (see Section 2.2) was used. On the other hand, a panel of eight experts from the food sectors of different European countries, with different backgrounds (i.e., academia and research centres) and expertise in NPD, evaluated all the ideas generated by the participants. These experts identified the dimensions (Table 2), from those obtained in the previous part (see Section 2.4.1), that were contained within each idea and their extent by scoring the dimensions identified in a continuous scale from 0 (“this dimension is not contained at all in this product idea”) to 10 (“this dimension is fully contained in this product idea”). Multiple factor analysis (MFA) was performed to determine the extent of agreement between the expert’s scores. As a high agreement was reached, the mean scores were used in subsequent analyses. The identification of the dimensions contained within each idea was necessary to explore whether there was a relationship between the importance of dimensions and their frequency of mention (see Section 2.4.3). In summary, the acceptability given by participants to each idea and the corresponding intensity for each dimension given by the experts were used to infer the importance attributed by the participants to each dimension.

To illustrate which dimensions had a greater influence on participant acceptability, a preference map [46] was created for each focus group. For that purpose, the experts’ mean scores per dimension and the mean acceptability of participants per idea were used. In addition, to overcome the limitations linked to the fact that the participants only scored the ideas generated within their focus group, an overall preference map was created. The results of the preference maps could be useful when selecting those ideas to scale up in the NPD process.

#### 2.4.3. Part 3: Dimensions’ Importance versus Dimensions’ Frequency

To determine whether there is a relationship between what consumers say (dimensions’ frequency) and what they like most about the ideas generated (inferred dimensions’ importance), an MFA between these two matrices was conducted. The dimensions’ frequencies were gathered from the reduced contingency table used to conduct the CA (see Figure 2). The dimensions’ importance matrix was built using dimensions (rows) and the inferred dimension’s importance for each participant, as explained in Table 1 (columns).

All statistical analyses were performed using XLSTAT software, version 2020.1 (2020) (Addinsoft, Paris, France).

## 3. Results

### 3.1. Part 1: Dimensions’ Identification and Their Frequencies

**Figure 3:**
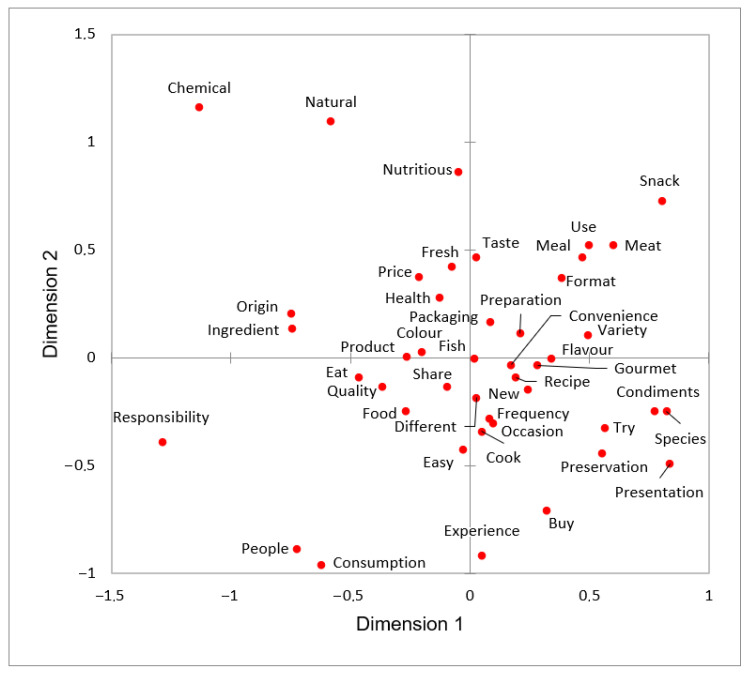
Multidimensional scaling of the 44 categories obtained using Alceste software, grouped by the first pseudo-triangulation process, and reduced by frequency of co-occurrence (Dimensions 1 and 2).

The automatic merging and reduction performed with the Alceste software allowed for a reduction in words from the 36,765 that had been in the initial corpus to only 747, retained for later analysis. After the first manual pseudo-triangulation process, 747 words were reduced to 186. Finally, from the 186 words obtained, only those whose diagonal values in the proximity matrix were greater than 50 were retained, as explained above, leaving 44 categories, as shown in the MDS plot (Figure 3).

The closer the categories are in the MDS plot, the higher the number of times those categories are mentioned together (co-occur). For example, the categories “ingredient” and “origin” (which grouped the word “local”) were repeatedly mentioned together, as exemplified in one quotation from a German participant: “Local ingredients are very important”. In addition, “quality” was frequently mentioned alongside the category “origin”, as shown in this quote by a Spanish participant: “It is the most determinant factor in the low quality, the origin of the fish”. As illustrated in another quotation from a focus group in France (i.e., “If it is quality fish, it is going to be expensive”), the categories “quality”, “fish”, and “price” (which grouped the word “expensive”) were frequently mentioned together. Interestingly, participants also frequently mentioned the categories “price” and “taste” in the same phrase; for example, “It is the price that decides, and the taste, of course” (a quote from a German participant).

As previously explained, some of the results obtained from the MDS were used to group the 44 categories into 12 dimensions through a second pseudo-triangulation process. For instance, the co-occurring categories “natural” and “chemical” in the MDS were grouped under the higher-order dimension “natural”. The final dimensions identified are listed in Table 2.

**Figure 4:**
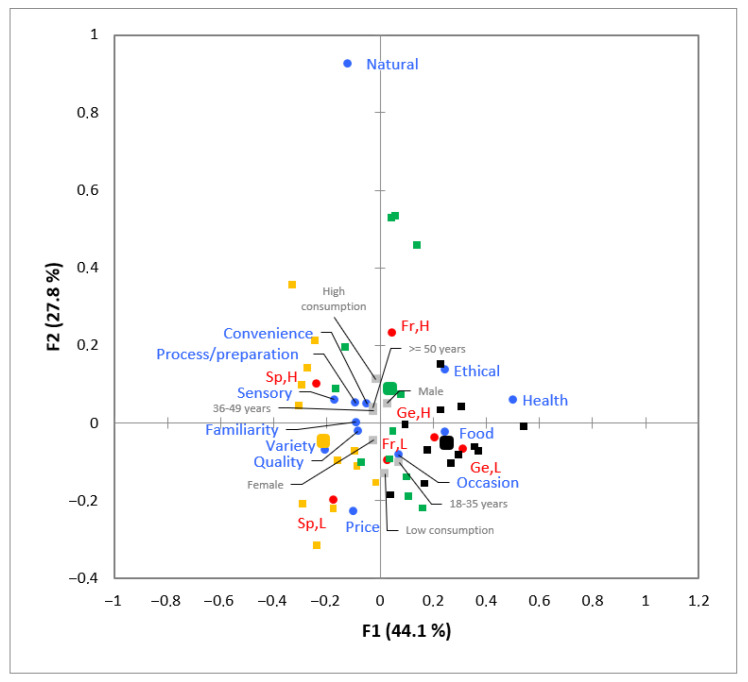
Correspondence analysis of the frequency of mention of the 12 dimensions and supplementary variables: high fish consumption (H); low fish consumption (L); black, German participants (Ge); yellow, Spanish participants (Sp); and green, French participants (Fr). Country totals are expressed in broader coloured points: light blue, dimensions; and light grey, supplementary variables.

The results of the CA performed on the 12 dimensions graphically displayed the contingency table with the dimensions’ frequency of mention and the supplementary variables (Figure 4), which allowed for the comparison between qualitative variables.

The German participants frequently mentioned the “food” and “health” dimensions. The dimension “ethical” was cited several times by both German and French participants. In the analysis, France was located closer to the “natural”, “occasion”, “convenience”, and “process/preparation” dimensions, but also to the “sensory”, “familiarity”, and “quality” ones, and all three were frequently cited by Spanish participants. Finally, the “price” and “variety” dimensions were also located closer to Spain.

### 3.2. Part 2: Dimensions’ Importance

Creative techniques (direct analogies and storyboarding) encouraged participants from all three countries to generate a pool of 112 new aquaculture fish product ideas. The differences between countries were observed. The Spanish participants generated a higher number of ideas (45 ideas), followed by the German (42) and French (25) participants. Small differences were found in the proportion of ideas generated in the focus groups within the same country. A slight difference in the number of ideas generated between the focus group with participants with high (23) and low (19) fish consumption was found in Germany. The 15 ideas with higher participants’ mean acceptability per country are shown in Appendix A (Table A1, Table A2 and Table A3). 

A high regression vector (RV) coefficient (0.7) between the expert scoring allowed for the use of their mean scores given to each dimension, together with the participants’ mean acceptability, to plot a preference map for each focus group. The RV coefficient measures the similarity between two matrices of quantitative variables or two configurations resulting from multivariate analysis [47]. The vector model was the best fit in all cases, with the sole exception of one focus group; that is, German consumers with high fish consumption, where the best-fitting model was the circular anti-ideal.

When comparing the preference maps between countries, the dimensions that had a greater influence on the acceptability scores for Spanish participants with high fish consumption were convenience, process/preparation, occasion, and variety. Spanish participants with low fish consumption attributed greater importance to ethical issues. French participants with high fish consumption placed more importance on health, quality, and natural aspects. In contrast, German participants focused on price, quality, naturalness, and ethical dimensions. Finally, participants from France and Germany with low fish consumption attributed more importance to ethical and price dimensions. It is noteworthy that in all focus groups with low fish consumption frequency, ethical issues played a relevant role (results not shown).

**Figure 5:**
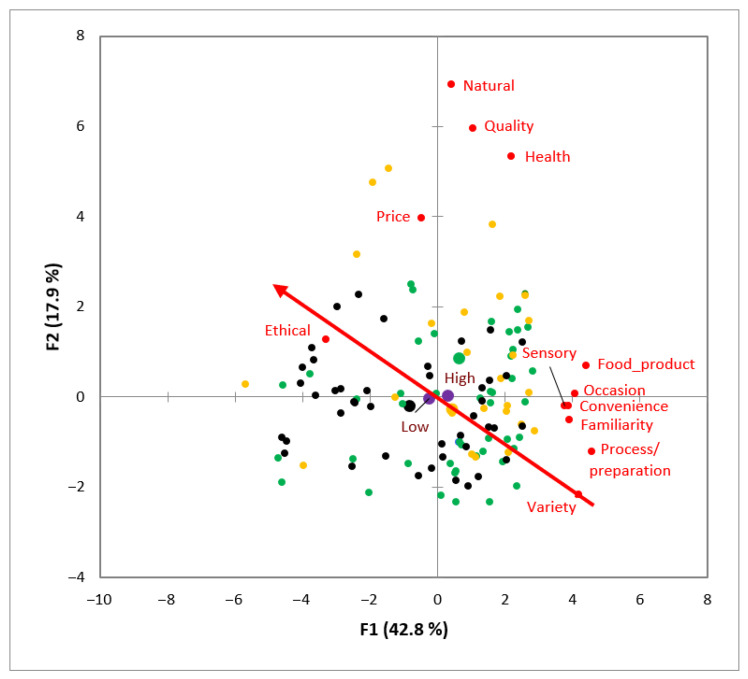
Overall preference map with the dimensions’ importance for participants and the supplementary variables: black, German participants; green, French participants; and yellow, Spanish participants. Country totals are expressed in broader coloured points: purple, consumers’ fish consumption (high or low). The red arrow indicates the vector model of preference.

The overall preference map allowed for the identification of the most important dimensions for the participants from all countries, and these were “natural”, “quality”, “ethical”, “price”, and “health” (Figure 5).

### 3.3. Part 3: Dimensions’ Importance versus Dimensions’ Frequency

The RV coefficient between matrices was 0.7, with most of the participants showing a significant correlation between the frequency of mentioning a specific dimension and the importance attributed to it. Some exceptions were found, namely one participant from Spain and four from France.

The results from the MFA showed that the dimensions with the weakest relationship between frequency of mention and importance were “food product”, “ethical”, “variety”, and “process/preparation” (graph not shown).

## 4. Discussion

### 4.1. Dimensions’ Identification and Their Frequencies

Although the Alceste software significantly reduced the number of words automatically and objectively [24], a larger screening of words was needed to facilitate the subsequent analysis. The manual merging through the first pseudo-triangulation process allowed us to perform a more thorough and goal-oriented reduction in the content. Therefore, the manual merging into high-order codes allowed for the maintenance of the information contained in the less frequently mentioned words, although this can possibly lead to a loss of information nuance [26]. Nevertheless, a loss of information and word misinterpretation are two major errors that can be mitigated during manual merging if the context of some words is considered during the pseudo-triangulation process. For example, if the word “know” is examined in isolation, it may be misinterpreted as “knowledge”. However, when considering the context, the majority (121 out of the 201 times the word “know” was mentioned) referred to the filler words “you know”; consequently, it was removed. Another example can be found in the semantic field grouping of words with related meanings. Atlantic pomfret fish would have been discarded if only the word frequency of mention had been used, as it was mentioned eight times in a single focus group. However, this information was retained, as it was grouped under the label “species”, along with other fish species (e.g., salmon or tuna). A more subjective association than a semantic field is the associative semantic field, in which the context gains special relevance. For example, the context served to group the word “shop” under the label “market”, as they were used as synonyms in more than 75% of the cases (e.g., “You could buy it at the bakery shop”). Finally, the context was also useful to group antonyms and homographs. For example, the word “orange” has a two-fold meaning (colour or fruit). In the present study, all mentions referred to the colour; therefore, it was grouped within the label “colour”. These examples highlight the importance of considering the context in which words have been said when interpreting qualitative data, allowing for the maintenance of relevant information while reducing the number of words, hence facilitating later analysis.

As demonstrated in the present study, discarding words in the first stages of the analysis based on frequency of mention normally means losing relevant content. However, at the later stage of analysis, when words are already grouped (automatically and/or manually), the frequency of mention can be used as a cut-off, which is normally used to simplify the information obtained. For example, after a manual merging process, Ares and Deliza [27] only retained those categories mentioned by more than 10% of the participants, while Ares et al. [28] used 5% as a cut-off point. Therefore, in the present study, a reduction based on the frequency of co-occurrence was performed at a later stage of the analysis, after automatic and manual merging.

The MDS results (Figure 3) plotted the strength of the connections between co-occurring categories [43] and allowed for the visualisation and interpretation of the structure of the data. Quality is one of the most important determinants in food purchasing, including fish and seafood product purchasing [48,49,50]. However, high-quality products at a “wrong” price are doomed to failure. Thus, interpreting words in isolation, such as “quality” and “price”, can lead to misleading conclusions, as the strong price–quality relationship has long been established in the extant literature (e.g., McConnell [51]). Indeed, earlier studies have found that consumers tend to use price as an indicator of food product quality [48,52]. Therefore, co-occurrence analysis becomes particularly relevant when interpreting qualitative data, as relationships between co-occurring words or concepts allow us to interpret them in their context [26].

Moreover, some of the results from the MDS were used when the categories were grouped through the second pseudo-triangulation process into dimensions because, as pointed out by Bazeley [43], considering co-occurrences is a critical aspect when generating higher-order codes (or dimensions). For this reason, co-occurrence analysis should be an essential step in any process involving grouping word or concepts into higher-order codes. Indeed, a word itself loses part of its idiosyncratic meaning when it cannot be related to any other term that gives meaning to it and places it in a specific context.

The results of the CA (Figure 4) performed on the 12 dimensions allowed for a deeper understanding of the associations between the supplementary variables in relation to the frequency of elicitation of the different dimensions. Frequencies of mention differed across countries, thus suggesting the existence of differences in the participants’ discourses [28]. For example, French and Spanish participants cited the “quality” dimension several times, as observed by Guerrero et al. [22] for French consumers when exploring the concept of innovation. Another example is the Spanish participants, who frequently mentioned the “price” dimension, similar to that reported in other studies, but for food and well-being [28]. Therefore, CA is seen as a valuable tool to graphically display the main findings of qualitative techniques, such as in the present focus groups.

The pseudo-triangulation principle, commonly applied to qualitative data analysis, requires considerable experience. Many studies have highlighted that researchers who conduct qualitative data analyses have at least two to five years of experience [18,22,28,53,54]. However, one of the advantages of the method proposed in this study for analysing qualitative data is that it does not require a high level of expertise, as the pseudo-triangulation process requires lower cognitive effort than traditional content analysis.

### 4.2. Idea Generation and Dimensions’ Importance

There were slight differences in the number of ideas generated by the participants between the countries. The Spanish participants generated the highest number of ideas, followed by the German and French participants. This data contrasts with the number of new fish products available in the market over the past two years, with Spain having the lowest product assortment range (445) compared with Germany (781) and France (803) [34]. In this sense, perhaps the German and French participants had difficulties proposing new ideas in a more saturated market. Another plausible explanation may be related to the relevant role that previous knowledge and past experiences play in creating new ideas [8,12]. As stated by Perkins (1988, cited in Witell et al. [8], p. 148), creativity consists of remembering already known ideas and reassembling them into novel ones. However, in our study, this relationship between already existing products (knowledge and experience) and creative ability (number of ideas generated) was not observed, probably because the culture and traditions related to fishing and fish, which are perhaps more prominent in Spain, as reflected by its higher fish consumption [55], play a role in shaping consumer attitudes in addition to knowledge and experience.

The participants registered their acceptance scores for every idea generated within their focus group; however, there were no clues indicating which reasons or dimensions had a higher influence on their scores (e.g., convenience or price). It may be that they only considered a few dimensions, the most salient ones for them, when giving their acceptance scores, similar to that described in the theory of planned behaviour (TPB), where only a few salient beliefs are the determinants of consumer behaviour [56]. Ideally, it would be preferable for consumers themselves to identify the dimensions contained within each idea. However, this additional step would have required more time, and it is unlikely that an average consumer could make this assessment objectively, without the interference of personal preferences or social desirability bias. Therefore, a group of experts was commissioned to identify which dimensions were contained within each idea and to what extent, assuming that the experts perceived the same dimensions as the participants did.

One of the main limitations of using a preference map per focus group is that the results obtained depend on the ideas generated within that session. In this sense, when a certain dimension is missing in a preference map, it does not mean that this dimension is not important, but rather that no ideas containing this dimension have been generated. To overcome this drawback, an overall preference map was created (Figure 5), independent of the ideas generated per focus group.

The dimensions with a greater influence on the consumers’ acceptance identified with preference maps define specific consumer segments (i.e., different countries and consumption frequencies). In future, the information obtained using preference maps (overall and per focus group) could be of the utmost importance for producers and marketers when selecting the ideas with greatest potential to be well received in the market, as it is likely that those ideas containing a dimension with great influence on consumer acceptance have greater potential for success in the market. In addition, it is highly advisable that the mean acceptance scores given by the participants to each idea be considered in the selection of the most promising ones. Therefore, it is likely that from those ideas gathered in Appendix A (Table A1, Table A2 and Table A3), the ones that are more convenient and varied will be more successful among Spanish consumers with high fish consumption; whereas the ideas with a strong, healthy and natural component will be more salient among French consumers. Nonetheless, the final selection of ideas to scale up in the NPD process will also depend on other aspects, such as economic and technical feasibility, company values, and target markets [57].

Despite the many benefits of involving consumers in the generation of ideas during NPD, co-creation also has its detractors. Some authors consider co-creation to have several limitations, including the idiosyncratic nature of consumer-generated ideas (i.e., personal preferences and nostalgic memories), rather than addressing societal, nutritional, or environmental challenges, and not ensuring innovativeness or technological breakthroughs [58]. Hence, it may be possible that consumers generate a product idea that, once available in the supermarket, they would not buy. Therefore, to measure the effectiveness of co-creation, it is necessary to conduct a validation study to determine whether the ideas generated by consumers would actually be more successful in the market.

### 4.3. Dimensions’ Importance versus Dimensions’ Frequency

The relationship between the participants’ frequency of mentioning a specific dimension and the importance attributed to it was high (RV coefficient 0.7). This finding shows that there is a strong correlation between what consumers say (frequency of mention) and what they like the most about the ideas generated (importance), thus confirming what Guerrero et al. [59] has already pointed out, i.e., that “the frequency of use of certain words may be related to the importance that it has for an individual”. It may be speculated that the correlation coefficient might have been higher if the analysis of the transcripts had been conducted per country, instead of in a combined manner. In addition, it is worth mentioning that a high correlation coefficient was obtained despite interference by experts, albeit this was necessary to identify the dimensions contained within each idea.

The dimensions with the weakest relationship between frequency of mention and importance found using MFA were “food product”, “ethical”, “variety”, and “process/preparation”. It is noteworthy that these dimensions grouped the greater number of categories each; that is, between five and six, along with the “sensory” dimension (Table 2). Therefore, it would have been appropriate to reduce the number of categories combined in the same dimension to reduce its variability. However, this explanation is not plausible for the “ethical” dimension, which only combined two categories. As some social conventions may constrain the expression of individuals’ opinions [13], the discrepancies between the frequency of mention and importance in the “ethical” dimension may have been caused by the social desirability bias. In other words, consumers may discuss ethical issues because interest in this topic is socially widespread; however, when scoring their acceptability for the ideas individually, they rate them lower than expected. Another possible explanation for the weak relationship between the frequency of mention and importance of some dimensions is that there was a discrepancy between the topics discussed and the ideas generated, although this is unlikely, because the ideas generated were the ones that gave rise to the subsequent discussion. In the hypothetical case that creative techniques would have been used after projective techniques (see Banović et al. [14]), it is likely that the agreement between the participants’ discourse and the ideas generated was higher. Nevertheless, the topics discussed in the focus group would have had a greater influence on the ideas generated. As stated by van Kleef and van Trijp [3], providing participants with information too early in the NPD “may kill the creativity”. To avoid this in the present study, the idea generation phase (creative) took place at the beginning of the focus group sessions.

### 4.4. Limitations and Suggestions for Future Research

One of the limitations of this study is that the content analysis was conducted by combining the transcripts from all countries together. In future studies, it would be interesting to explore whether the results might have been richer if the analyses had been conducted within each country individually, as country-dependent nuances (e.g., the use of different words, the syntactic structure of the different languages, or the unequal importance of specific dimensions) may have been lost when the analysis was conducted for all the countries together.

Another interesting area of study would be to explore new ways to simplify the methodological approach presented; although it has been proven successful, it required less experience, and it was relatively economical in terms of cost, it was very time-consuming.

Finally, an additional limitation of the study is that Alceste software is not open access, a fact that may hinder its accessibility to potential users.

## 5. Conclusions

The qualitative data analysis methodology described in this study is deemed useful for drawing key conclusions from qualitative research, thus offering an alternative to traditional content analysis. The combination of statistical qualitative research software (Alceste), word frequencies, context, and co-occurrence analysis, although more time-consuming than a simple semantic approach, provided rich and valid information, close to the real outcomes generated during the qualitative phase. In particular, the long time needed to prepare the data, conduct the pseudo-triangulation processes, and carry out statistical analysis to identify the most salient dimensions behind the participants’ discourse is the major drawback of the methodological approach presented in this study. Conversely, the strength of the approach described is the lower level of expertise required, as grouping words according to the guidelines provided required less experience and cognitive effort than the deep semantic content analysis normally applied to traditional qualitative data analysis. However, both approaches are complementary.

The use of co-creation with consumers to generate new product ideas, with the consequent benefits of incorporating their voices during the early stages of the NPD process, allows us to escape from traditional firm-centred new product design. Focus groups were a useful tool for applying co-creation techniques; in particular, a combination of creative and projective techniques. Creative techniques allowed participants to generate not only food product ideas, but also product concepts, which are particularly useful when designing food packaging or advertising campaigns. For example, “more sustainability in fish-breeding” is a product concept generated by the participants that highlights their concern about sustainability and leads to the consideration of adding sustainable aquaculture quality labels (e.g., ASC). The combination of creative and projective techniques proved to be very useful, allowing for the capture of not only cognitive, but also unconscious individual desires, thus providing complementary information.

Finally, the present study has proven that there is a strong correlation between what consumers say (frequency of mention) and what they attribute more importance to. This finding could be of the utmost importance when interpreting qualitative data, as the frequency of mention could be used to infer the importance for individuals. Thus, consumers talk more about the topics they like or care about the most. As the frequency of mention has been proven to be correlated with participants’ acceptance, future studies can replicate the approach presented here without the assistance of experts, thus simplifying the method, as one of the main drawbacks associated with the scoring of dimensions is the requirement for a group of experts.

## Figures and Tables

**Table 1:** Fictitious example of the calculation of the dimensions’ importance for participant 1.

	Experts’ Mean		Acceptability	Dimension’s Importance	
Ideas	Sensory	Price	Participant 1	Sensory	Price
Idea 1	2	6	9	18 *	54
Idea 2	7	4	5	35	20
			∑	53 **	74

* The importance of the sensory dimension for idea 1 was calculated by multiplying the experts’ mean scores for sensory dimension and the consumer’s (participant 1) acceptability for this idea. The same procedure was conducted for all dimensions, ideas, and participants. ** The dimension’s importance was then computed as the sum of all individual importances (for all the ideas); higher values in the sum indicates the higher importance of that dimension for participant 1. In this fictitious example, the dimension price is more important for participant 1 than sensory one.

**Table 2:** Dimensions formulated by the grouping of the 44 categories through the second pseudo-triangulation process.

Dimension	Categories
Health	Health, nutritious
Process/preparation	Product, preparation, condiments, cook, ingredient, recipe
Sensory	Colour, experience, flavour, gourmet, taste, try
Quality	Quality, fresh, origin, preservation
Price	Price, buy
Familiarity	Frequency, use
Natural	Natural, chemical
Food_product	Consumption, eat, fish, meat, food, meal
Variety	Variety, different, format, new, presentation, species
Convenience	Convenience, easy, snack
Ethical	Responsibility, packaging
Occasion	Occasion, people, share

## Data Availability

The data presented in this study are available on request from the corresponding author.

## Appendix A

**Table A1:** Ranking of ideas with higher participant acceptability in France.

Ranking	New Product Idea	Total Score ^1^	Average Score ^2^	SD ^3^
1	Fish brochettes for grilling, heathy appetizers	46.0	7.7	1.0
2	Aquaculture fish without additives, without chemicals	44.0	7.3	1.8
3	Salad with fish for self-service	43.5	7.0	1.9
4	Fish salad with biodegradable packaging, scannable to know the ingredients	42.0	6.7	2.3
5	Fishballs for snacking/appetizer	42.0	6.5	2.2
6	Mousse to spread on bread	42.0	6.3	3.0
7	Fish cooked with Reunion Island flavours and tastes	41.5	6.0	1.6
8	Box for snacking with assorted fishes	40.0	6.0	2.3
9	Ready sauce with fish	40.0	5.2	2.7
10	Fish mousse without bones, with dill, only fish	39.0	4.2	2.4
11	Frozen or chilled dishes, gourmet, designed by chefs	39.0	6.5	2.7
12	Fish dish with innovative sauce and fish-shaped packaging	38.5	6.4	2.8
13	Fish cooked in a natural way	38.0	6.3	2.3
14	Fish carpaccio	36.0	6.0	2.5
15	Uncommon fish species crumbed and of high quality	36.0	6.0	2.3

^1^ Sum of all participants’ acceptability within a focus group, scored on a scale from 1 (I very much dislike this fish product idea) to 10 (I like this fish product idea). Minimum score: 6, maximum: 60. ^2^ Participants’ mean acceptability of every idea: sum of all participants’ acceptability divided by the number of participants within each focus group (6). ^3^ SD: standard deviation.

**Table A2:** Ranking of ideas with higher participant acceptability in Germany.

Ranking	New Product Idea	Total Score ^1^	Average Score ^2^	SD ^3^
1	New packaging for frozen fish (e.g., bag instead of using foil)	55.0	9.2	1.0
2	Recyclable packaging (second use)	52.0	8.7	2.0
3	High-grade convenience products, local, quick	51.0	8.5	2.1
4	Avoiding problems (e.g., storage life, no shell, no fishbone, etc.)	47.0	7.8	2.2
5	Product with healthy ingredients (e.g., for crumb coating) like protein bars	46.0	7.7	2.7
6	Combination of different fish tastes and textures	45.0	7.5	1.8
7	New fish convenience products	44.0	7.3	2.5
8	A whole fish (fillable, already filleted, etc.)	44.0	7.3	2.4
9	No-waste burger	44.0	7.3	2.5
10	Packaging not made of plastic or aluminium	41.0	6.8	3.9
11	Fish with more power/enriched/all necessary ingredients contained	37.0	6.2	3.3
12	Product containing only fish, no other animal products	36.0	6.0	3.5
13	Canned fish with new supplements (e.g., lentils)	35.0	5.8	2.6
14	Fish-to-go (patty for burger, etc.), for microwave	35.0	5.8	2.4
15	Fish that does not taste fishy	32.0	5.3	2.4

^1^ Sum of all participants’ acceptability within a focus group, scored on a scale from 1 (I very much dislike this fish product idea) to 10 (I like this fish product idea). Minimum score: 6, maximum: 60. ^2^ Participants’ mean acceptability of every idea: sum of all participants’ acceptability divided by the number of participants within each focus group (6). ^3^ SD: standard deviation.

**Table A3:** Ranking of ideas with higher participant acceptability in Spain.

Ranking	New Product Idea	Total Score ^1^	Average Score ^2^	SD ^3^
1	With flavours, to mix with a small sauce bag (fine herbs, olive oil, pepper, mustard, garlic, and lemon)	57.0	9.5	0.8
2	Seabass, seabream, meagre in dices or crumbs, frozen, and boneless	56.0	9.3	1.2
3	Fish hamburgers	51.0	8.5	1.4
4	Tasty and boneless fish products	50.0	8.3	1.2
5	Coloured spaghetti surimi to decorate and add flavour	49.0	8.2	2.8
6	Fish tray (such as cheese tray), two fish species in the same tray, fresh	49.0	8.2	1.2
7	Fillets, cubes, smoked, in brine	49.0	8.2	1.2
8	Seabass fillets	48.0	8.0	2.0
9	New formats for children (stars, trapezoids, triangles)	48.0	8.0	2.5
10	Sushi with nice flavours	47.0	7.8	1.9
11	Fish with sauce preparation, tasty, high quality	47.0	7.8	1.7
12	Fish with flavour of other things (e.g., octopus + cooked potato, anchovies + chip potato)	44.0	7.3	3.3
13	Fish products with an extra healthy component	44.0	7.3	1.2
14	Small fish pieces with intense flavour	39.0	6.5	2.4
15	Different fish assortment, shellfish, different tastes	36.0	6.0	2.2

^1^ Sum of all participants’ acceptability within a focus group, scored on a scale from 1 (I very much dislike this fish product idea) to 10 (I like this fish product idea). Minimum score: 6, maximum: 60. ^2^ Participants’ mean acceptability of every idea: sum of all participants’ acceptability divided by the number of participants within each focus group (6). ^3^ SD: standard deviation.
